# Protocol for a Sepsis Model Utilizing Fecal Suspension in Mice: Fecal Suspension Intraperitoneal Injection Model

**DOI:** 10.3389/fmed.2022.765805

**Published:** 2022-05-12

**Authors:** Takumi Tsuchida, Takeshi Wada, Asumi Mizugaki, Yoshitaka Oda, Katsuhide Kayano, Kazuma Yamakawa, Shinya Tanaka

**Affiliations:** ^1^Division of Acute and Critical Care Medicine, Department of Anesthesiology and Critical Care Medicine, Faculty of Medicine, Hokkaido University, Sapporo, Japan; ^2^Department of Cancer Pathology, Faculty of Medicine, WPI-ICReDD, Hokkaido University, Sapporo, Japan; ^3^Department of Emergency Medicine, Osaka Medical and Pharmaceutical University, Takatsuki, Japan

**Keywords:** sepsis, mice, cytokine, cecal ligation and puncture, peritonitis, inflammation

## Abstract

**Background:**

Various animal models of sepsis have been developed to optimize sepsis treatment. However, therapeutic agents that were successful in animal models were rarely effective in human clinical trials. The cecal ligation and puncture (CLP) model is currently the gold standard for sepsis studies. However, its limitations include the high variability among researchers and the difficulty in comparing animals with different cecum shapes and sizes. In this study, we established a protocol for the creation of a simple and accessible sepsis rodent model using fecal suspensions that minimized differences in technical effects among researchers and individual differences in animals.

**Methods:**

A mouse model of sepsis using fecal suspension intraperitoneal injection (FSI) was created using fresh stool excreted within 24 h. The collected fresh stool was dissolved in saline solution and filtered. The obtained fecal suspension was injected intraperitoneally into the mice. Moreover, fecal suspensions with different concentrations were prepared, and the survival rates were compared among the fecal suspensions for each concentration. To assess the validity of the FSI as a sepsis model, CLP and FSI with similar mortality rates were compared pathologically, physiologically, immunologically, and bacteriologically. Histopathological comparison was evaluated by hematoxylin-eosin and Gram staining of the parenchymal organs. Physiological evaluation was performed by comparing the respiratory rate, body temperature, and blood gas analysis results. Immunological assessment was performed using multiplex analysis. Bacteriological comparisons were performed by culturing ascites fluid.

**Results:**

The FSI model increased mortality in proportion to the fecal suspension concentration. The mortality rate was reduced with antibiotic administration. In various comparative experiments conducted using the FSI and CLP models, both models showed findings consistent with sepsis. Furthermore, the FSI model showed less variability among the individuals in each test.

**Conclusion:**

This is the first detailed and accurate report of a protocol for creating a sepsis model using fecal suspension. The FSI model is a minimally invasive and accessible sepsis rodent model. Its clinical validity as a sepsis model was proven via histological, physiological, microbiological, and immunological evaluation methods. The FSI model minimizes individual differences between mice and helps to conduct accurate studies after the onset of sepsis.

## Introduction

Sepsis is a common disease with a mortality rate of 28–48% (1–3). To develop superior sepsis treatments, various studies using animal models of sepsis have been conducted. However, even if an animal model demonstrates the efficacy of a therapeutic agent, it rarely leads to success in human clinical trials. The ideal animal model used to develop sepsis therapeutics should mimic the course of human diseases. However, there are major differences among the current animal sepsis models, and there is no optimal model for drug discovery of sepsis thus far. There are many possible causes for this setback. To date, a variety of sepsis models have been developed (4–13). Each sepsis model has its own specific advantages and disadvantages, which have been discussed in previous reviews (6–8, 14, 15).

At present, the cecal ligation and puncture (CLP) model is considered the gold standard for sepsis studies (8, 9). The main feature of the CLP model is that it recreates the hemodynamic and metabolic phases of human sepsis (8); further, apoptosis of selected cell types and the host immune response also resemble the course of human sepsis (16, 17). However, it suffers from such limitations as the high variability among researchers (18) and the difficulty in making comparisons among animals with different cecum shapes and sizes (4). Another known model of sepsis using fecal suspensions has been reported in mice (4), rats (11), and sheep (12, 13). However, its position as a sepsis model is not well established because their preparation methods are not clearly described (11) or are complicated (4, 12, 13).

In this study, we devised a simple and easy animal model of sepsis using fecal suspensions that minimizes the effects of differences in techniques used by researchers, and individual differences in animals. We prepared two different concentrations of fecal suspensions (thin and thick) to confirm the effects of antimicrobial agents. The survival rates were compared according to various concentrations of each fecal suspension. The validity of the model was evaluated using bacteriological, pathological, physiological, and immunological methods.

## Materials and Methods

### Ethics Statement

All experiments were conducted in accordance with the Hokkaido University Animal Experiment Regulations. The present study followed international, national, and/or institutional guidelines for humane animal treatment and complied with relevant legislation from the Institutional Ethical Review Board of Hokkaido University (Approval number: 20-0163).

### Experimental Animals

Seven-week-old Institute of Cancer Research male mice were obtained from Japan SLC Inc., (Hamamatsu, Japan). Mice were specific-pathogen-free and weighed 32–34 g.

### Housing and Husbandry

All animals were housed and treated in accordance with the guidelines for performing animal experiments at Hokkaido University. Following an acclimation period of a minimum of 3 days in the animal breeding quarters, the animals were subjected to experimentation. Mice were housed in a facility with a stable room temperature and humidity (24 ± 2°C, 0–20%) and a regular light/dark cycle (12 h of light from 6 a.m. to 6 p.m.). The mice were fed a standard diet (Labo MR Stock^®^, Japan Nosan Corporation, Kanagawa, Japan) and given free access to water from a water bottle placed above the cage. The bottom of the cage was covered with paper matting (Paper clean^®^, Japan SLC Inc., Hamamatsu, Japan) that was changed every few days to keep the cage clean. The number of mice in each cage was limited to five.

### Experimental Procedures (Creating a Model Mouse)

The CLP model was created using the procedure of Rittirsch et al. (19) with adipose tissue resection (20). A 10 mm incision was made in the midline of the shaved and sterilized abdomen. A 21-gauge needle was used for the puncture, and the ligation site was half the distance between the distal pole and the base of the cecum. Silk thread (2-0) was used to ligate the cecum, and a 4-0 nylon thread was used to suture the peritoneum and skin. Anesthesia was administered with ketamine (125 mg/kg) and xylazine (10 mg/kg), and buprenorphine 0.05 mg/kg was administered for adequate analgesia. After surgery, 1 mL of saline was administered subcutaneously for fluid resuscitation. To prevent post-surgical infection, the mice were placed in the supine position in the cage until the effect of the anesthesia wore off. The cage was warmed with a heater to prevent hypothermia after surgery. Postoperative antibacterial agents of imipenem/cilastatin at a dose of 25 mg/kg twice daily for 3 days (21) were used to prevent infections. The first antibiotic administration was performed 2 h after surgery.

**FIGURE 1:**
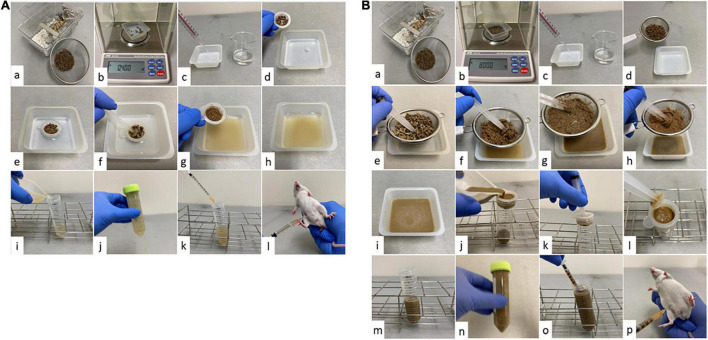
Procedure for creating the fecal suspension intraperitoneal injection (FSI) model. **(A)** FSI model with thin fecal suspension. (a) Collect the required amount of feces from the cage. Spread enough paper matting to prevent urine from mixing with feces. Eliminate as much non-fecal waste as possible and be careful not to let water drip from the water bottle. (b) Weigh out 400 mg (450 mg for making 30 mg/mL) from the collected feces. (c) Weigh out 40, 20, 10, 15, or 8 mL of saline solution into a tray, depending on the concentration of fecal suspension to be prepared. (d) Putting feces on a Falcon^®^ 70 μm cell strainer. (e) Soak the feces in the saline solution through the filter and macerate it thoroughly. (f) Filter the feces using a Falcon^®^ 70 μm cell strainer and grind stick (we used a syringe pusher as a grind stick). (g) Grind the feces well until it becomes a paste (until the feces is no longer gritty). After filtration, drain the water from the paste feces well. (h) The obtained fecal suspension is impurity-free and can be aspirated without resistance with a 25-gauge needle. (i) Transfer the fecal suspension to a container that can be stirred. (j) Shake the tube well to mix sufficiently. Step (j) is performed once for each procedure (k). (k) Immediately after Step (j), aspirate 1 ml of fecal suspension with a syringe. (l) Administer 1 ml of fecal suspension intraperitoneally to the mice with a 25-gauge needle. **(B)** FSI model with thick fecal suspension. (a) Collect the required amount of feces from the cage. Spread enough paper matting to prevent urine from mixing with feces. Eliminate as much non-fecal waste as possible and be careful not to let water drip from the water bottle. (b) Weigh out 8,000 mg from the collected feces. (c) Weigh out 80, 60, 53, 40, or 27 mL of saline solution into a tray, depending on the concentration of fecal suspension to be prepared. (d) Putting feces on a tea strainer. (e) Soak the feces in the saline solution through a tea strainer and macerate it thoroughly. (f) Filter the feces using a tea strainer and grind stick (We used a syringe pusher as a grind stick). (g) Grind the feces well until it becomes a paste (until the feces is no longer gritty). (h) After rough filtration, drain the water from the paste feces well. (i) The obtained fecal suspension is full of impurities and still cannot be aspirated with a 25-gauge needle. (j) Pour the fecal suspension onto a Falcon^®^ 70 μm cell strainer placed over a 50 ml tube. (k) Use a grind stick when necessary to promote filtration. (l) If the filter is clogged, remove the residue as needed. (m) The obtained fecal suspension is impurity-free and can be aspirated without resistance with a 25-gauge needle. (n) Shake the tube well to mix sufficiently. Step (n) is performed once for each procedure (o). (o) Immediately after Step (n), aspirate 1 mL of fecal suspension with a syringe. (p) Administer 1 ml of fecal suspension intraperitoneally to the mice with a 25-gauge needle.

The details of the FSI model preparation are shown in Figure 1; the thin and thich fecal suspensions are shown in Figures 1A,B, respectively. The cage was replaced 24 h before surgery to remove the old stool. Two types of suspensions were prepared, the thin and thick fecal suspension, depending on whether antimicrobial agents were used after surgery. Filtration of the thick fecal suspension was performed in two stages: coarse filtration using a commercial 630 μm tea strainer (product number DH7086, Kai Industries Co., Ltd., Tokyo, Japan) and fine filtration using a Falcon 70 μm Cell Strainer (product number 352350; Corning, New York, United States). Thin fecal suspension was prepared by suspending 400 mg of feces in 40, 20, 10, and 8 mL of saline solution, or 450 mg of feces in 15 mL of saline solution. The concentrations of the prepared thin fecal suspensions were 10, 20, 30, 40, and 50 mg/mL. Thick fecal suspensions were prepared by suspending 8,000 mg of feces in 80, 60, 53, 40, and 27 mL of saline solution. The concentrations of the prepared thick fecal suspensions were 100, 133, 151, 200, and 296 mg/mL. The prepared fecal suspensions were intraperitoneally administered to each mouse at a volume of 1 mL. Buprenorphine 0.05 mg/kg was administered for analgesia. Since there was enough water in the fecal suspension, fluid resuscitation was not performed as in postoperative CLP. As with CLP, the postoperative antibacterial agents were imipenem/cilastatin at a dose of 25 mg/kg twice a day for 3 days. The first antibiotic administration was performed 2 h after the injection of fecal suspension.

### Experimental Outcomes

The survival rate was evaluated for 14 days by setting the day 0 from 0 to 24 h after surgery and continuing observation until day 14. Experiments comparing the CLP and FSI models [experiments (i) through (iv) below] were performed before the mice began to die, that is, 20 h after surgery.

### Study Design

We compared the survival rates of the FSI models at various severities. Survival rates were verified in groups of 10 animals each. Next, the following experiments were performed using the FSI model (i.e., 133 mg/mL FSI model), which had a mortality rate similar to that of the CLP model: (i) histopathological comparison of parenchymal organs; (ii) bacteriological comparison by ascites culture; (iii) physiological comparison by respiratory rate, body temperature, and blood gas analysis; and (iv) immunological comparison by multiplex analysis by Luminex^®^ 100/200™ (R&D Systems Inc., Minneapolis, MN, United States). Comparison (i) was performed between groups of three animals each. Comparisons for (ii) to (iv) were performed between groups of five animals each. The control group consisted of three animals each for (i) and five animals for (iii) and (iv).

### Allocating Animals to Experimental Groups

Survival comparison experiments and comparison experiments between the CLP model and FSI model [experiments (i) to (iv)] were conducted independently on different days. To reduce the number of mice used, the same mice were used for experiments (i) and (iii) and for experiments (ii) and (iv). Mice were randomly assigned to the required number and groups.

### Experimental Procedures (Comparison of Cecal Ligation and Puncture and Fecal Suspension Intraperitoneal Injection Models)

Histopathological comparisons were performed using the kidneys, spleen, lungs, and liver. Organs were taken 20 h after surgery from three animals each from the CLP and FSI models and placed in a 10% formalin neutral buffer solution. For euthanasia prior to organ removal, ketamine 250 mg/kg and xylazine 20 mg/kg were used. These were almost twice the doses used for surgical anesthesia. Tissue sections were then prepared from these organs using a microtome. The tissue sections were assessed using hematoxylin and eosin (HE) staining. Gram stain was added to the lung, kidney, spleen, and liver sections. In addition, post-tonsillectomy and post-adenoidectomy hemorrhage stain was added to the kidney sections. Gitter staining was performed for the liver sections. Elastica–Masson staining was performed on the lung sections for examination.

Bacteriological comparisons were assessed by culturing the ascites fluid 20 h after surgery. In addition, two sets of culture tests were performed on the cecal feces of mice that were not treated with antibiotics as controls and on the fecal suspension used to create the FSI model. Ascites fluid, collected after injecting 5 mL of saline into the abdominal cavity of mice with a 25-gauge needle and agitated well, was extracted using a clean syringe. Since multiple lavages could increase the errors, once the 5 mL of saline was injected, the saline was collected as much as possible for bacteriological evaluation. During the collection of the ascites fluid, the body surface of the shaved mice and the instruments used were disinfected with alcohol to prevent contamination, and the entire procedure was a clean operation. For ascites collection, 250 mg/kg ketamine and 20 mg/kg xylazine were administered subcutaneously to the backs of the mice as anesthesia. Samples were cultured on Nissui Plate Sheep Blood Agar (Nissui Pharmaceutical Co., Ltd., Tokyo, Japan) and DHL agar (Becton Dickinson and Company, Tokyo, Japan) for capneic incubation and Anaero Columbia Agar with rabbit blood and PEA/BBE agar (Becton Dickinson and Company, Tokyo, Japan) for anaerobic culture. After incubation, the number of bacteria was determined semi-quantitatively based on the number of colonies. Bacterial species were identified by mass spectrometry using a MALDI Biotyper^®^ (Bruker Japan K.K., Kanagawa, Japan).

Respiratory rate and body temperature were compared for physiological evaluation. Blood gas analysis was performed. Respiratory rate was measured visually, and body temperature was determined as the average of three measurements taken with a high-performance cutaneous infrared thermometer (product number CTD711, Citizen Co., Ltd., Tokyo, Japan). Cardiac blood from mice anesthetized with ketamine 250 mg/kg and xylazine 20 mg/kg was collected using a heparin-coated syringe. Blood samples were analyzed using the ABL800 FLEX^®^ (Radiometer Medical ApS, Kobenhavn, Denmark).

Immunological evaluation was performed by multiplex analysis using Luminex^®^ 100/200™ (R&D Systems Inc., Minneapolis, MN, United States). Blood from mice was collected in the same way as in the physiological evaluation, and the obtained blood was centrifuged at 2,000 g for 10 min to separate the plasma. Plasma was subjected to multiplex analysis with a Luminex^®^ 100/200™ using MILLIPLEX^®^ MAP kit (Merck Millipore Corporation, Darmstadt, Germany) as a reagent.

### Statistical Analysis

All data are expressed as the median and standard error (SE). Data were analyzed using the Mann–Whitney test using IBM SPSS software (version 25; IBM Japan, Tokyo, Japan). Statistical significance was set at *P* < 0.05.

## Results

### Survival Rate

**FIGURE 2:**
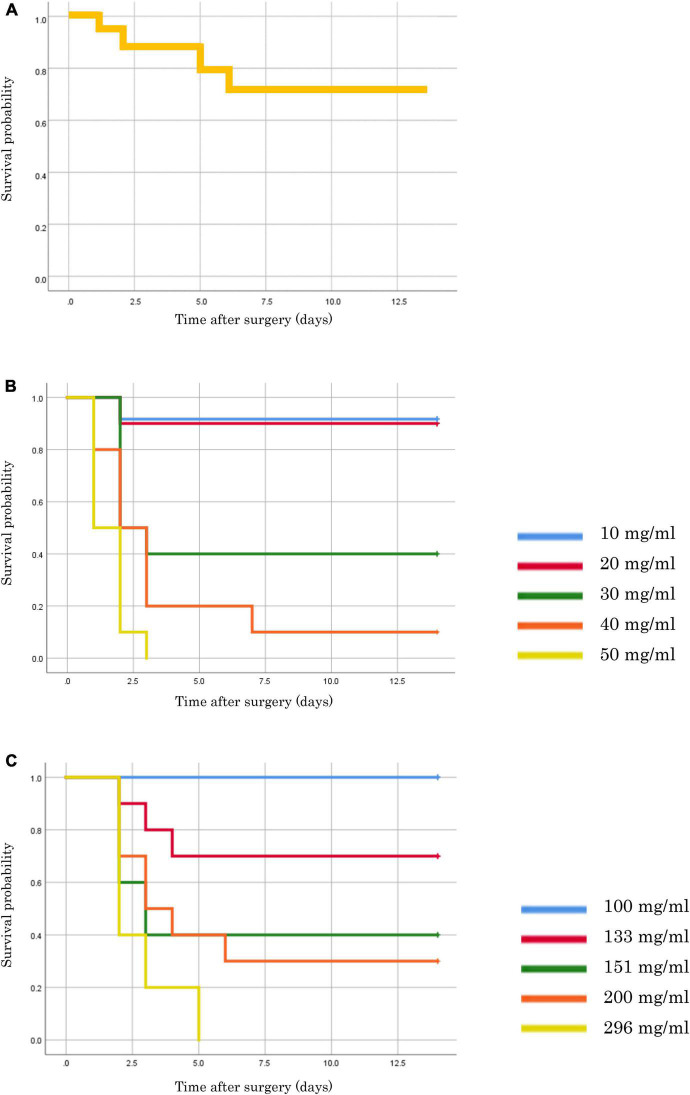
Survival rates of each model mouse. **(A)** CLP model. **(B)** FSI model of thin fecal suspension (without antimicrobial administration). **(C)** FSI model of thick fecal suspension (with antimicrobial administration).

The survival curves for each model are shown in Figure 2. Fourteen CLP models were created using 15 mice, as one mouse died due to anesthesia. The survival rate of the CLP model after 14 days was 71.4% (Figure 2A). The FSI model showed an increased mortality rate in proportion to the concentration of fecal suspension. Without antimicrobial administration, mice often died early (up to 3 days) after fecal suspension injection (Figure 2B). In the FSI model with antibiotic administration, the mice did not die from fecal suspensions of up to 100 mg/mL. Therefore, it was necessary to create the thick fecal suspensions (100–296 mg/mL) to validate the mortality rate following antimicrobial use. Results from the model with a thick fecal suspension showed that antibiotics reduced mortality and prolonged survival (Figure 2C).

### Bacteriological Evaluation

**FIGURE 3:**
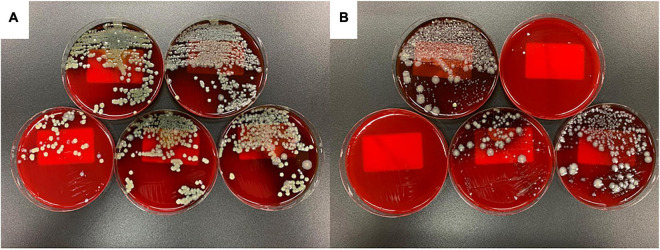
Results of ascites culture of FSI and CLP models 20 h after surgery. Results, 24 h after the start of capneic incubation with Sheep Blood Agar **(A)** FSI model, **(B)** CLP model.

Figure 3 shows the results of bacterial culture of ascites at 20 h after the onset of peritonitis. In the FSI model, 10∧3 colony forming units (CFU)/mL or more of multiple bacterial species were cultivated from all animals (Figure 3A and Table 1). However, in the CLP model, there were animals with negative cultures or very small numbers of bacteria, and individual differences were large (Figure 3B). Although there was a slight difference in the bacterial species between the stool culture in the cecum and the fecal suspension culture, no significant difference was observed in ascites cultures between the FSI and CLP models. *Stenotrophomonas maltophilia* was detected at an equal rate in the ascites culture of the FSI model, which was the most significant difference between the FSI and CLP models (Table 1).

**TABLE 1:** Results of bacterial species identification in ascites culture.

	FSI model					CLP model					Feces in the cecum		Fecal suspension	
	1	2	3	4	5	1	2	3	4	5	1	2	1	2
*Enterococcus gallinarum*										⊚	⊚	⊚	⊚	
*Enterococcus faecalis*	○	⊚	○		○	⊚	○		⊚		⊚			
*Lactobacillus* species			○		○	⊚	△		⊚	⊚	⊚	⊚		
*Escherichia coli*		○		○	○		△		○		⊚	⊚	⊚	⊚
*Bacteroides vulgatus*						⊚	○		○	⊚	⊚	⊚	⊚	⊚
*Lactococcus garvieae*			○										⊚	⊚
*Proteus mirabilis*		○			△	⊚				△			⊚	⊚
*Klebsiella oxytoca*						○				⊚				
*Stenotrophomonas maltophilia*	⊚	⊚	○	⊚	⊚									

*Ascites was evaluated with samples taken 20 h after the onset of peritonitis. Results of identification of bacterial numbers and species in ascites culture of 5 FSI models and 5 CLP models. Bacterial numbers are evaluated by colony forming unit (CFU) per 1 mL and displayed in three stages (⊚:10∧5 CFU/ml or more, ○:10∧3 or more and less than 10∧5 CFU/ml, △:less than 10∧3 CFU/ml). One of the CLP model sample had no bacterial growth. Bacterial identification tests were also performed on stool collected from the cecum of two non-antibiotic mice and fecal suspension (two samples) used to create the FSI model.*

### Histopathological Evaluation

**FIGURE 4:**
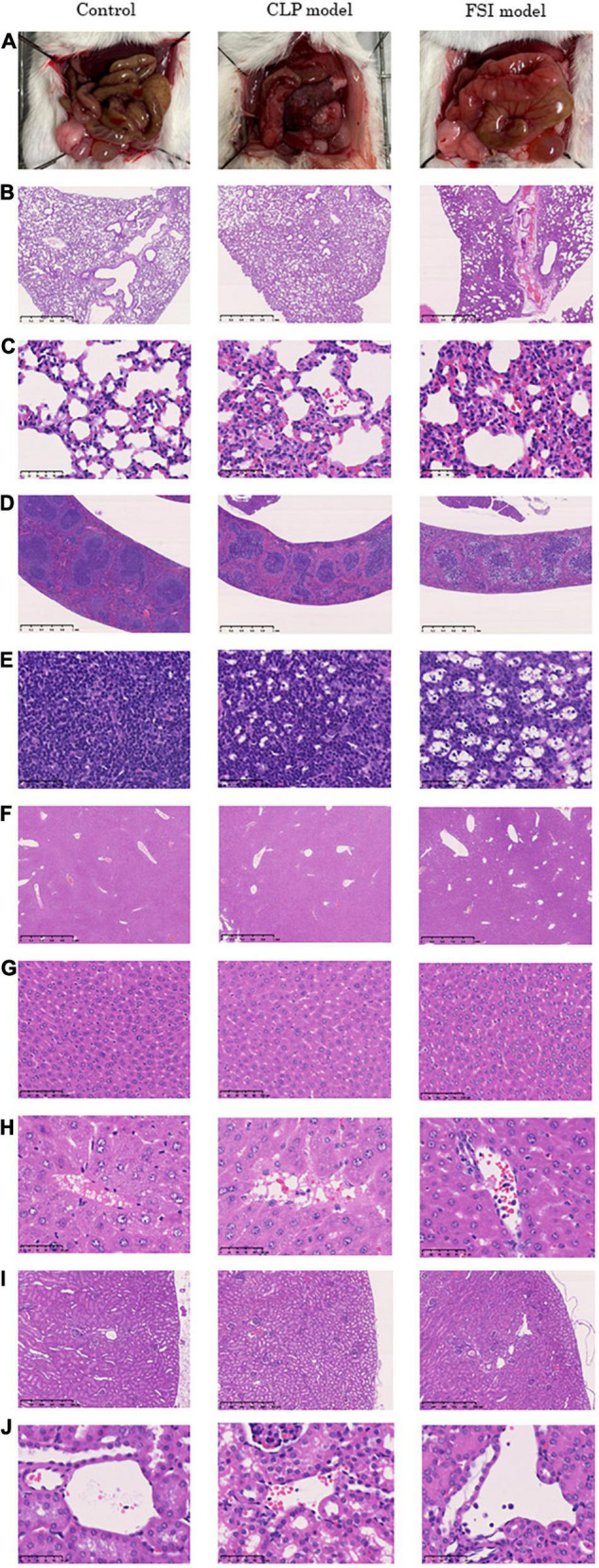
Histopathological findings 20 h after onset of peritonitis in FSI model, CLP model and control cases. **(A)** Microscopic findings of the abdominal cavity. **(B)** 2.5×(Objective lens) lung tissues. **(C)** 40×(Objective lens) lung tissues. **(D)** 2.5×(Objective lens) spleen tissues. **(E)** 40×(Objective lens) spleen tissues. **(F)** 2.5×(Objective lens) liver tissues. **(G)** 20×(Objective lens) liver tissues. **(H)** 40×(Objective lens) liver tissues. **(I)** 5×(Objective lens) kidney tissues. **(J)** 40×(Objective lens) kidney tissues.

The pathological findings at 20 h after surgery for the FSI and CLP models are shown in Figure 4. The microscopic findings of the abdominal cavity are shown in Figure 4A. The FSI model showed significant edema of the intestine, while the CLP model showed necrosis in addition to edema of the intestine. In the lungs, congestion and neutrophil infiltration were observed in both groups. Neutrophil infiltration into the alveolar space was not observed in any case, and no traces of pneumonia were noted in any of the lung samples (Figures 4B,C). In the spleen, hemophagocytosis was found both in the FSI and CLP model cases, but not in the control case (Figures 4D,E). Histopathological findings in the lung and spleen supported that the FSI model caused systemic inflammation that was of the same intensity or more severe than that in the CLP model cases. In the liver, the sinusoids were dilated, reflecting congestion findings (Figure 4G). There were no remarkable morphological changes in the kidneys (Figures 4I,J). In particular, microthrombi indicating disseminated intravascular coagulation were not detectable in the glomerulus, and centrilobular degeneration of hepatocytes, which reflects shock, was not found in any case. A few neutrophil infiltrates were found in the portal veins, central veins of the liver, and renal small vessels in the FSI model case, while a few neutrophil infiltrates were found in the central veins of the liver in the CLP model case (Figures 4H,J). Bacteria were not detected in any organ in any of the cases.

### Physiological Evaluation

The respiratory rate, body temperature, and blood gas analysis results at 20 h after surgery for FSI and CLP models are shown in Table 2. Both the FSI and CLP models exhibited tachypnea, hypothermia, and hypoglycemia. In addition, hyperchloremia was observed in both models, which was thought to be due to the bolus administration of saline. In addition, the FSI model had severe metabolic acidosis, which was different from the CLP model. The CLP model showed no significant acidosis findings compared to the control group. In the CLP model, the standard error was greater than that in the FSI model for most variables.

**TABLE 2:** Results of the respiratory rate, body temperature, and blood gas analysis 20 h after surgery for FSI and CLP models.

	Control	FSI model	CLP model
Respiratory rate (/min)	136 ± 4.476	204 ± 11.123*	160 ± 6.849*
Body temperature (°C)	37.0 ± 0.085	36.1 ± 0.044*	36.0 ± 0.164*
pH	7.241 ± 0.0034	6.974 ± 0.0110*^#^	7.148 ± 0.0300
pCO_2_ (mmHg)	57.2 ± 2.276	82.4 ± 3.991*	63.3 ± 3.523
pO_2_ (mmHg)	49.1 ± 4.161	30.6 ± 2.724	42.8 ± 2.705
K (mmol/L)	3.7 ± 0.061	4.6 ± 0.171*	3.9 ± 0.203
Na (mmol/L)	148 ± 0.219	150 ± 0.456^#^	154 ± 1.308*
Cl (mmol/L)	118 ± 0.867	127 ± 0.780*	125 ± 2.028*
Hemoglobin (g/dL)	13.0 ± 0.461	14.3 ± 0.614	13.7 ± 1.141
Hematocrit (%)	39.9 ± 1.385	43.8 ± 1.846	42.1 ± 3.449
HCO3 (mmol/L)	23.4 ± 0.999	18.2 ± 1.089*^#^	25.0 ± 1.592
Base excess (mmol/L)	−2.9 ± 0.946	−12.0 ± 1.102*^#^	−2.8 ± 1.837
Glucose (mg/dL)	298 ± 8.112	125 ± 11.229*	112 ± 17.035*
Lactate (mmol/L)	2.8 ± 0.118	3.2 ± 0.481	2.1 ± 0.275

Data presented as median l’ standard error. *Significantly different from the control group (p-value < 0.05). ^#^Significantly different from the CLP group (p-value < 0.05).

### Immunological Evaluation

**FIGURE 5:**
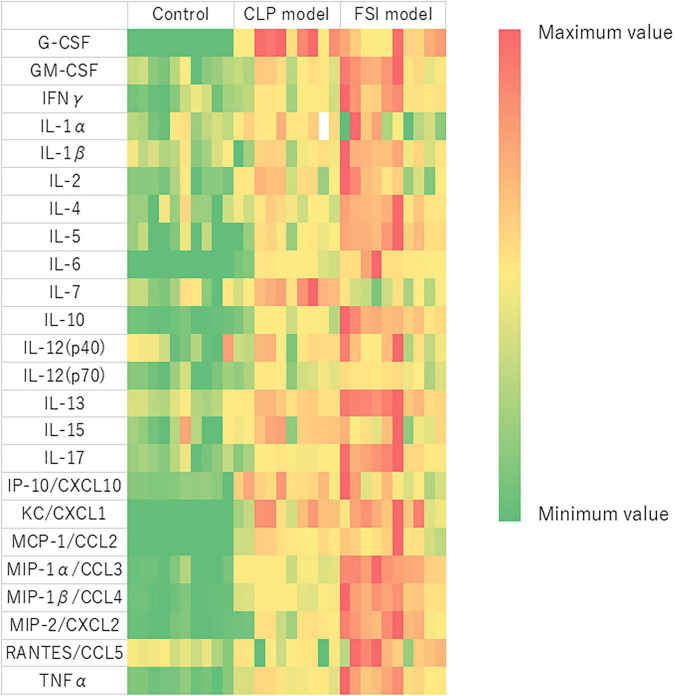
Heatmap analysis of cytokine and chemokine measurements performed by multiplex analysis for FSI and CLP models. Blood was evaluated with samples taken 20 h after the onset of peritonitis. Heat map showing relative evaluation of cytokine and chemokine values. The maximum value of each variable is red, the minimum value is green, and the intermediate value corresponds to the gradation scale from red to green. G-CSF, Granulocyte-colony stimulating factor; GM-CSF, Granulocyte macrophage colony-stimulating factor; IFNγ, Interferon gamma; IL, Interleukin; IP, Interferon gamma-induced protein; CXCL, C-X-C motif chemokine ligand; KC, Keratinocyte-derived chemokines; MCP, Monocyte chemotactic protein; CCL, C-C motif chemokine; MIP, Macrophage inflammatory protein; RANTES, Regulated on activation, normal T cell expressed and secreted; TNF, tumor necrosis factor.

Comprehensive measurements of inflammatory-related cytokines and chemokines revealed a high inflammatory response in both the FSI and CLP models at 20 h after surgery (Figure 5 and Table 3). In the FSI model, a strong inflammatory reaction was observed. Because of this difference, levels of IL-1β and RANTES were high only in the FSI model, while IL-1α and IL-7 levels were high only in the CLP model. No increase was observed in IL-9 levels in any model. Although it differs slightly depending on the measurement parameters, the standard error of the FSI model was smaller overall.

**TABLE 3:** Cytokine and chemokine measurements performed by multiplex analysis in FSI and CLP models 20 h after onset of peritonitis.

	Control	FSI model	CLP model
G-CSF	46.9 ± 5.0	89451.3 ± 734.2*	92031.9 ± 629.0*
GM-CSF	54.1 ± 7.3	196.7 ± 10.6*^#^	107.0 ± 23.4*
IFNγ	3.3 ± 1.0	37.6 ± 1.5*^#^	11.7 ± 13.1*
IL-1α	66.0 ± 13.5	73.3 ± 16.9	136.4 ± 51.3*
IL-1β	5.8 ± 0.6	17.9 ± 1.4*^#^	8.1 ± 2.7
IL-2	3.2 ± 0.8	12.9 ± 1.5*	11.4 ± 3.1*
IL-4	0.6 ± 0.1	3.0 ± 0.1*^#^	1.1 ± 0.4*
IL-5	1.9 ± 0.7	22.2 ± 1.2*^#^	8.4 ± 2.7*
IL-6	4.6 ± 0.7	17553.0 ± 1126.7*^#^	2720.4 ± 14972.8*
IL-7	4.9 ± 1.2	6.1 ± 4.6^#^	26.8 ± 0.9*
IL-9	151.7 ± 35.0	160.7 ± 141.9	93.4 ± 52.3
IL-10	10.4 ± 2.1	1594.9 ± 16.4*^#^	133.1 ± 347.4*
IL-12(p40)	12.2 ± 4.0	21.8 ± 3.1*	23.4 ± 5.1*
IL-12(p70)	12.7 ± 4.1	94.8 ± 6.9*^#^	48.5 ± 12.7*
IL-13	40.5 ± 5.3	560.8 ± 28.4*^#^	168.5 ± 67.0*
IL-15	47.1 ± 22.1	139.9 ± 18.1*	182.7 ± 24.0*
IL-17	3.0 ± 0.6	73.6 ± 1.1*^#^	8.2 ± 14.6*
IP-10/CXCL10	94.1 ± 11.7	408.4 ± 300.0*	484.7 ± 61.2*
KC/CXCL1	158.2 ± 25.0	18761.0 ± 2137.1*	17657.4 ± 2032.9*
MCP-1/CCL2	58.1 ± 4.5	6618.7 ± 468.3*	5992.5 ± 1182.5*
MIP-1α/CCL3	42.3 ± 4.9	599.1 ± 5.0*^#^	138.4 ± 58.5*
MIP-1β/CCL4	68.7 ± 5.8	4123.3 ± 26.7*^#^	424.8 ± 667.3*
MIP-2/CXCL2	32.0 ± 15.4	4732.8 ± 192.7*^#^	966.2 ± 871.9*
RANTES/CCL5	47.7 ± 3.7	185.0 ± 9.0*^#^	65.7 ± 42.0
TNFα	6.4 ± 1.0	113.5 ± 5.2*^#^	39.7 ± 23.2*

Data presented as median ± standard error. All units are picogram per milliliter (pg/ml). *Significantly different from the control group (p-value < 0.05). ^#^Significantly different from the CLP group (p-value < 0.05). G-CSF, Granulocyte-colony stimulating factor; GM-CSF, Granulocyte macrophage colony-stimulating factor; IFNγ, Interferon gamma; IL, Interleukin; IP, Interferon gamma-induced protein; CXCL, C-X-C motif chemokine ligand; KC, Keratinocyte-derived chemokines; MCP, Monocyte chemotactic protein; CCL, C-C motif chemokine; MIP, Macrophage inflammatory protein; RANTES, Regulated on activation, normal T cell expressed and secreted; TNF, tumor necrosis factor.

## Discussion

We established a protocol for a murine sepsis model (FSI) using fecal suspensions made from excreted feces. Comparison between the FSI and CLP (gold-standard) models physiologically, pathologically, bacteriologically, and immunologically indicated that the FSI model showed almost similar changes as the CLP model. In addition, the FSI model showed less variation than the CLP model. These results suggest that the FSI sepsis model may be better than the conventional model.

The rodent CLP model is known to present with typical symptoms of sepsis or septic shock, such as hypothermia, tachycardia, and tachypnea (16), and it is also associated with hypoglycemia (22). Therefore, the results of the physiological evaluations in the FSI and CLP models were consistent with those observed in sepsis. Pathological evaluation revealed neutrophil infiltration into the parenchymal organs in both the CLP and FSI models. Although Gram staining of organs did not identify the bacteria and could not directly prove bacteremia, bacteriological evaluation confirmed that similar bacteria were cultivated in the ascites culture in both models. As a result of these detailed comparisons, both the FSI and CLP models were considered as being consistent with sepsis. Immunological evaluation using Luminex^®^ showed that all cytokines and chemokines, except IL-7, RANTES, and IL-9, were elevated in both the FSI and CLP models (Figure 5 and Table 3). In particular, the FSI model showed higher levels of inflammation with subsequent higher levels of anti-inflammatory cytokines, such as IL-4 and IL-10, than the CLP model. RANTES is elevated in sepsis and promotes neutrophil infiltration into the lungs (23). In this study, only the FSI model showed elevated RANTES levels, supporting the sepsis-like characteristics of the FSI model rather than the CLP model. IL-7 is a potent anti-apoptotic cytokine that is essential for lymphocyte survival and expansion (24, 25). Only the CLP model showed significantly higher IL-7 levels in our study, but there was no correlation between plasma IL-7 levels and the severity of sepsis (26). Therefore, although there are some differences between the FSI and CLP models, which may be due to the difference in time phase, the results are immunologically consistent with sepsis.

The major features of the FSI model are that peritonitis can be easily created without the need for anesthesia or surgical procedures and that mice models with uniform severity of sepsis can be developed. The results obtained by physiological, bacteriological, and immunological evaluations showed that there was little variation among individuals in the FSI model, suggesting that the influence of individual differences in mice during model creation could be minimized. A previous study demonstrated a cecal slurry preparation protocol similar to the current FSI model. As shown in this study, this protocol requires preparation of cecal slurry with glycerol-PBS, freezing of the cecal slurry at −80°C, and thawing (4). These suggest that the procedures for cecal slurry are considerably more complicated than for the FSI. The fact that most of the other models using fecal suspensions require surgical intervention (12, 13) also implies that the FSI has a stronger advantage in terms of the simplicity of its creation process. In addition, sacrificial deaths of multiple mice are needed just to obtain the cecal slurry (4). Thus, we believe that the simplicity and uniformity of the FSI model allowed us to reduce the strain on the mice.

Existing animal sepsis models can be divided into three categories: exogenous administration of a toxin, exogenous administration of a viable pathogen, or alteration of the animal’s endogenous protective barrier (14). Injection of specific toxins or pathogens alone are problematic in that they are different from the clinical pathology, although accurate information with few errors can be obtained. On the other hand, the model that induces sepsis by changing the endogenous protective barrier of animals, represented by the CLP model, is problematic in that it is difficult to obtain genuine information due to large individual differences; however, it is the closest to the clinical pathology of sepsis. The FSI model facilitates the homogenous development of peritonitis due to multiple causative organisms, much like a model injecting a specific toxin or pathogen. In the FSI model, the detection rate of *S. maltophilia* in ascites culture was very high, and the concentration was also high (Table 1). *S. maltophilia* is an emerging multidrug-resistant, global opportunistic pathogen (27). Although it is not a highly pathogenic pathogen, it is an important in-hospital pathogen associated with bloodstream mortality (28, 29). In the FSI model, administration of antibiotics may have increased the occupancy of *S. maltophilia* because there was no influx of new feces from the intestinal tract.

In the CLP model, the severity of disease varies greatly depending on the degree of fecal outflow from the cecum perforation site, the degree of filming (localization) of the perforation site, or the degree of necrosis of the distal cecum (14, 30–32). This makes it difficult to examine the pathophysiology of sepsis, especially in periods other than the hyperacute phase. In the present study, adipose tissue was resected to prevent localized inflammation (20), but bacteriological evaluation showed that there were individual mice with negative ascites cultures and very low bacterial growth, thus prompting the hypothesis that adipose tissue resection during a CLP procedure cannot completely prevent localized abscess formation. Localization of peritonitis due to abscess formation has been a major problem in the CLP model as drugs that have proven to be beneficial during experiments using the CLP model with abscess formation can be ineffective in clinical sepsis treatment (14). The FSI model can overcome these problems inherent in the CLP model.

### Limitations

This study had some limitations. The experimental results were obtained at a single institution, and the protocol and experimental results of the FSI model need to be verified at other institutions. The results may vary depending on the facility, operator, condition of the stool, and degree of filtration and agitation of the stool suspension. It may be necessary to arrange the fecal dosage and filtration method, depending on the facility, to create mice with the desired severity. However, in any case, it is expected that there will be less variability when using the FSI model than with the CLP model.

As regards the physiological evaluation, the blood gas analyzer is designed for human blood samples; thus, the results may be inaccurate. The results of the immunological evaluation may also be affected by the administration of the anesthetic agent. During the bacteriological evaluation, there were samples with many species of bacteria, and it is possible that not all bacteria were identified. The FSI model progresses to severe disease more rapidly than the CLP model because a large amount of the pathogen is administered intraperitoneally at once. Therefore, even if the comparison is between models with the same mortality rate over the same time course, it may reflect a different pathological condition.

## Conclusion

In this study, we presented a protocol for an easy-to-create animal model of sepsis, the FSI model, and proved its clinical validity as a sepsis model by histological, physiological, bacteriological, and immunological methods. We believe that the FSI model, which can minimize individual differences among animals, will help produce new findings in sepsis research and contribute to the improvement of clinical sepsis outcomes.

## Data Availability Statement

The raw data supporting the conclusions of this article will be made available by the authors, without undue reservation.

## Ethics Statement

All experiments were conducted in accordance with the Hokkaido University Animal Experiment Regulations. The present study followed international, national, and/or institutional guidelines for humane animal treatment and complied with relevant legislation from the Institutional Ethical Review Board of Hokkaido University (Approval number: 20-0163).

## Author Contributions

TT contributed to the research conception, analysis, experimentation, and manuscript preparation. TW contributed to the research concept and oversaw the entire study. AM contributed to the performance of the experiments. YO and ST contributed to the provision of histopathological photographs and pathological evaluation. KY contributed to immunological evaluation through sample measurements. KK contributed to the revision of the intellectual content. All authors have read and approved the final version of the manuscript prior to submission.

## Conflict of Interest

The authors declare that the research was conducted in the absence of any commercial or financial relationships that could be construed as a potential conflict of interest.

## Publisher’s Note

All claims expressed in this article are solely those of the authors and do not necessarily represent those of their affiliated organizations, or those of the publisher, the editors and the reviewers. Any product that may be evaluated in this article, or claim that may be made by its manufacturer, is not guaranteed or endorsed by the publisher.
